# Metronidazole concentration in the bloodstream following its topical application, at different concentration levels, on experimental skin wounds during healing by secondary intention^1^

**DOI:** 10.1590/s0102-865020190010000004

**Published:** 2019-02-14

**Authors:** Cláudia Paraguaçu Pupo Sampaio, Maria de Lourdes Pessole Biondo-Simões, Lilian Cristine Teixeira Trindade, Márcia Olandowski, Jorge Eduardo Fouto Matias

**Affiliations:** I Fellow PhD degree, Postgraduate Program in Surgical Medicine, Universidade Federal do Paraná (UFPR), Curitiba-PR, Brazil. Conception and design of the study, manuscript writing.; II Full Professor, Department of Surgery, and Permanent Instructor, Postgraduate Program in Surgical Medicine, UFPR, Curitiba-PR, Brazil. Design of the study, analysis of data, manuscript writing, final approval the version to be published.; III Associate Professor, Department of Statistics, Pontifícia Universidade Católica do Paraná (PUC-PR), Curitiba-PR, Brazil. Discussion of the project, and choice of evaluation methods.; IV Associate Professor, Department of Surgery, and Permanent Instructor, Postgraduate in Surgical Medicine, UFPR, Curitiba-PR, Brazil. Technical procedures.

**Keywords:** Wound Healing, Metronidazole, Skin Absorption, Administration, Topical, Rats

## Abstract

**Purpose:**

To characterize qualitatively and quantitatively the absorption of metronidazole solution, in greater concentrations and for longer periods, when applied topically to an experimental open skin wound model.

**Methods:**

An open skin wound, 2 cm in diameter and total skin thickness was prepared, under anesthetic, in the dorsal region of 108 Wistar rats weighing between 300 and 350 grams. The animals were allocated to groups of 18 animals in accordance with the concentration of metronidazole in the solution to be applied daily to the wound. In the control group (CG), 0.9% sodium chloride solution was used for application, and in the experimental groups (GI, GII, GIII, GIV and GV) metronidazole solution at 4%, 6%, 8%, 10% and 12%, respectively, was applied. After 3, 7 and 14 days of treatment. Blood samples collected through cardiac puncture were examined for the existence or non-existence of metronidazole, using high performance liquid chromatography (HPLC). Detected metronidazole values were compared statistically within each group (temporal analysis 3 days X 7 days X 14 days) and between the groups that used topical metronidazole (4% X 6% X 8% X 10% and 12%) using the Kruskal-Wallis test, considering a statistical significance of 95% (p<0.05).

**Results:**

Metronidazole was detected in all the samples at all times in all the groups in which topical metronidazole was applied to the wounds. Characteristically, there was no significant difference between the doses obtained within each group over time (3 days X 7 days X 14 days) GI=0.461; GII=0.154; GIII=0.888; GIV= 0.264 and GV=0.152. In the evaluation between groups, a similar degree of absorption was found after 3 days (p=0.829) and 14 days (p=0.751).

**Conclusion:**

The serum concentration of metronidazole that was achieved was not influenced by the concentration of the solution applied to the skin wound, with similar extend, or by the duration of the application.

## Introduction

 Metronidazole (1-beta-hydroxyethyl-2-methyl-5-nitroimidazole) is an active antibiotic against protozoa (Entamoeba histolytica, Giardia lamblia)^1^ ^,^ ^2^ and against obligate anaerobic bacteria, including *Bacteroides fragilis, Clostridium, Fusobacterium, Peptococcus, Peptoestreptococcus, Eubacterium, Helicobacter, Campilobacter fetus, Espiroquetas* and *Gardnerella vaginalis* ^3^. 

 Due to its good tissue penetration, it can reach therapeutic levels in bones, joints and cerebrospinal fluid. It is effective on septicemia and bacterial endocarditis caused by anaerobes^3^ ^-^ ^7^. 

 The topical use of metronidazole, in gel or solution form, has been proposed to control the odor of infected wounds^8^. The deodorizing effect is justified by the eradication of anaerobic infection^9^.

 Furthermore, metronidazole can have a favorable influence on skin healing, accelerating epithelialization, reducing repair time^10^ ^-^ ^13^. In general, the results show the favoring of early re-epithelization^12^ ^-^ ^15^, although the granulation and reepithelization time are apparently not influenced by the form of the doses used. However, very low concentration may not be sufficient and very high concentration may have a toxic effect, with both having a harmful effect on the healing process^10^ ^-^ ^16^.

 Regarding topical use on skin wounds, questions are asked concerning the potential effects following its probable absorption, both systemically, in the distance, and locally, on the skin lesion itself.

 The major challenge when evaluating the systemic effect and presence of metronidazole, regarding its topical application, is the lack of standardization of formulations. The kinetics of percutaneous absorption of topical formulations has shown that concentration is not the most important factor, but rather the vehicle. In this respect, the topical application of a cream would favor systemic absorption less than a lotion, and this in turn less than a gel^17^. Nevertheless, metronidazole gel at 25%, when used in periodontal pockets, did not induce high plasma concentrations^18^.

 The purpose of this study was to analyze the influence of the topical application of solutions with different concentrations of metronidazole (4%, 6%, 8%, 10% and 12%) on skin wounds, and serum levels over longer periods (3, 7 and 14 days) of application.

## Methods

 The research project was conducted following the analysis and approval of the Ethics Committee on the Use of Animals of the Pontifícia Universidade Católica do Paraná. The project was approved under Protocol Number 654/2012. The study adhered to the guidelines of Federal Law 11.794 and the recommendations of the Brazilian College of Animal Experimentation. 

 The sample consisted of 108 male rats (*Rattus norvegicus albinus, Rodentia mammalia*) of the Wistar lineage. The rats were 110 days old and weighed between 300g and 350g. The animals were kept in the laboratory for an acclimatization period in individual cages, with free access to water and standard feed for the species and under controlled environmental conditions of lighting (12-hour light/dark cycle), temperature (18 ± 2ºC) and relative air humidity (55 ± 15%). 

 Under anesthetic obtained from 80 mg/kg ketamine and 8 mg/kg xylazine with intramuscular administration, following trichotomy and antisepsis, in the dorsal region, a circular area of skin two centimeters in diameter, demarcated by the cutting edge of a metal punch, was excised, fully exposing the dorsal muscular fascia. There was no need for hemostasis and the area was left exposed. Immediately following the procedure, the animals were given a 10mg/kg dose of dipyrone by intramuscular (IM) administration for analgesic purposes.

 Six groups of 18 animals each were randomly formed according to the concentration of the metronidazole solution used in the daily treatment of their wounds. In the control group (CG), metronidazole solution was not used. Instead, they were given a 0.9% sodium chloride solution. In all the other groups, an aqueous solution of pure metronidazole was used, diluted in a final solution with the necessary amount, 40mg/kg (4%) in Group I (GI), 60mg/kg (6%) in Group II (GII), 80mg/kg (8%) in Group III (GIII), 100mg/kg (10%) in Group IV (GIV) and 120mg/kg (12%) in Group V (GV).

 All the wounds that were prepared in all the groups of animals were treated using the same protocol^19^. The lesions were washed on a daily basis with 0.9% sodium chloride solution and the crusts removed. This was followed by an application of 0.3 ml metronidazole solution, diluted in accordance with each group. 

 In each group, blood samples were taken to determine the presence of metronidazole in their systemic circulation at three times: the third, seventh and fourteenth days after the preparation of the skin wound. This subdivided each group into three subgroups according to the day when the blood samples were collected. The samples were collected by cardiac puncture, under anesthetic, with the removal of three milliliters of blood, stored in flasks containing ethylenediaminetetraacetic acid (EDTA), placed in a thermal box^20^ and immediately sent to the Analytical Center of the Technological Institute of Paraná (TECPAR), where the samples were centrifuged and frozen for later analysis. Following the cardiac puncture, the animals were submitted to euthanasia with a lethal dose of intra-peritoneal sodium thiopental (120mg/kg). 

 The analysis of the presence of metronidazole in the blood was conducted using high performance liquid chromatography (HPLC) through ionic pairing with two Nova Pak® columns with 10% carbon and an acetonitrile mobile phase at 15%. To calibrate the Agilent® chromatograph, model 1100, the allometric extrapolation was considered, determining the metabolization curve of the metronidazole through gavage of the solution with 80 mg/ml in other animals and collecting blood after 30, 60 and 120 minutes. Initially, the product applied to the wounds, in a quantity containing 40 mg of metronidazole, was compared at 10.6 mg of pure metronidazole to confirm identity. Both were diluted in 100 ml of methanol/H_2_O (1:1) and applied to the columns (10uL) at a flux rate of 0.4 ml/min, a temperature of 30ºC and wavelength of 272 nanometers (nm). The approximate concentration of the standard metronidazole was calculated from the areas obtained by injecting the standard sample of metronidazole (10 mg/L), this being 104.327 units of absorbance per second (uA.s). All the blood samples collected and frozen from all the study groups (4%, 6%, 8%, 10% and 12%) and respective subgroups (3 days, 7 days and 14 days subgrups) were prepared and analyzed using chromatography in the same conditions as described above. The concentration was evaluated by the behavior of the area using the following equation: *concentration = X factor area of proportionality + linear coefficient of the line*. The area was calculated by the chromatograph with the aid of Chem Station software*.*

 The area results were described by means, medians, minimum values, maximum values and standard deviations. To compare the groups on each evaluation day and to compare the times of evaluation within each group, the non-parametric Kruskal-Wallis test was used. P-values <0.05 indicated statistical significance. The data were analyzed using the Statistical v.8.0 computational program.

## Results

 The chromatogram that was obtained with the blood sample taken 120 minutes after the gavage of metronidazole at 4%, in accordance with the calibration method of the chromatograph, confirmed the sensitivity of the apparatus for the detection of metronidazole in blood samples. The presence of the same chromatographic peak confirmed the identity of the substance used in the sample.

 Standard metronidazole and the test substance (metronidazole used in the study) had identical physico-chemical behavior on the third, seventh and fourteenth days, at different mobile phase polarities (acetonitrile), showing the same retention time at the same conditions of flow, temperature and were length, identifying the test substance as metronidazole. 

**Figure 1:**
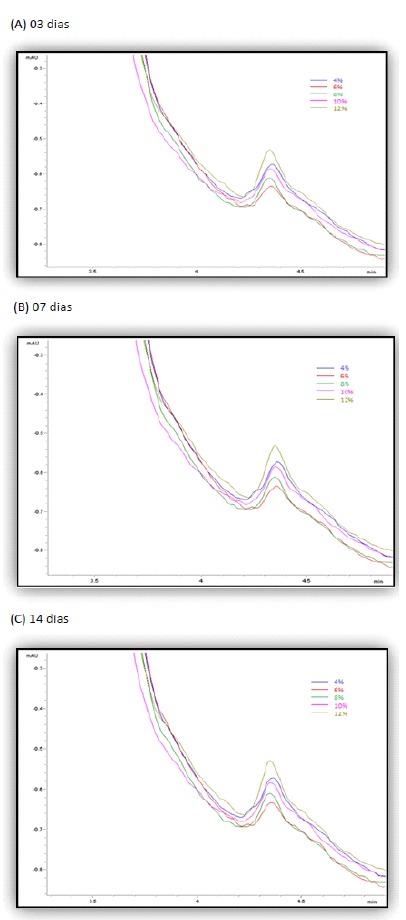
Chromatographic traces (HPLC) with the individual detection curves of metronidazole in the blood samples of the animals that were given topical administrations of the metronidazole solution at 4%, 6%, 8%, 10% and 12% for 3 days (**A**), 7 days (**B**) and 14 days (**C**).

 It was possible to record, with security and precision, the presence of metronidazole in the bloodstream of every animal whose dorsal skin wound was exposed only to the topical application of the product in all the concentrations used in this study (4%, 6%, 8%, 10% and 12% groups) and at every evaluation time (03, 07 and 14 day subgroups) (Figure 1). In the control group, all the chromatographic analyses of the samples were negative for the detection of the metronidazole substance. 

 In the quantitative chromatographic analysis, comparing the mean values of area obtained using the different concentrations of the metronidazole solution, it was possible to observe, characteristically, the invariability of the doses obtained from the bloodstream irrespective of the concentration of metronidazole in the solution used for the daily topical applications. Therefore, there was no quantitative variation of the blood dosages of metronidazole obtained from all the experiment groups, both in terms of concentration of metronidazole in the solution used for the applications (4% to 12%) and the time for which the respective solutions were used (3 days to 14 days) (Table 1).

**Table 1:** Absorption: intergroup statistical comparisons (4% X 6% X 8% X 10% X 12%) and intragroup comparisons (3 X 7 X 14 days) of the mean areas in uA.s obtained using high performance liquid chromatography (HPLC).

**TIME** **(days)**	**GI=4%**	**GII=6%**	**GIII=8%**	**GIV=10%**	**GV=12%**	
	**Area**±**sd (uA.s)**	**Area**±**sd (uA.s)**	**Area**±**sd (uA.s)**	**Area**±**sd (uA.s)**	**Area**±**sd (uA.s)**	**p**
**03 days**	0.4818±0.1245	0.6557±0.4347	0.5038±0.1698	0.5872±0.1083	0.5715±0.3045	0.829
**07 days**	0.6567±0.2103	0.9907±0.2983	0.5083±0.3432	0.5083±0.012	0.2810±0.0433	NA*
**14 days**	0.6340±0.4634	0.7056±0.5252	0.5083±0.0603	0.6853±0.3324	0.6535±0.3029	0.751
**p**	0.461	0.154	0.888	0.264	0.152	

*NA= Not Applied. Statistical comparison unfeasible due to loss of sample.

## Discussion

 As the treatment of wounds and the topical use of active principles improves, with a wide variety of effects and purposes, it is necessary to gain in-depth knowledge to discover the possible systemic and local consequences, whether beneficial or not, of the topical and prolonged application of active substances on different bleeding areas that cannot be isolated.

 As the subject in question is antimicrobial, with well-known actions against various microorganisms and of very important therapeutic value, the local use of metronidazole on skin wounds raises the pertinent questions regarding safe topical with regard to some aspects. Would the molecule be effectively absorbed by the bleeding area, permeating the bloodstream to the point of interfering with commensal microbiota at a distance? If such consistent plasma levels are reached, it could be asked which method is actually responsible for the potential benefits on the treated wound. Furthermore, would there be a real risk of toxic effects due to frequent and prolonged, albeit topical, use? 

 It is important to know which minimum concentration would be capable of facilitating healing and the maximum concentration at which a toxic action might occur. It is of fundamental important to define whether the metronidazole is absorbed in the area of the lesion when it is used topically. 

 The wide variation of metronidazole concentrations for topical use at the experimental level is an additional difficulty. There are studies that report the effects of metronidazole on healing using 20mg/kg^16^, 50mg/kg^14^, 108 mg/kg^10^, 160mg/kg^13^ and 180mg/kg^12^.

 This study evaluated the presence of metronidazole, in the blood following its exclusive topical use on uninfected wounds, seeking to establish a parameter of absorption and standard behavior according to the concentration and the time during which the substance was used.

 To evaluate absorption, the same method (topical), the same volume (0.3ml) and different concentrations (4%, 6%, 8%, 10% and 12%) were used. Evaluations were made of blood samples at different times (third, seventh and fourteenth day). It is important to determine whether topical metronidazole, capable of facilitating healing^10^ ^,^ ^12^ ^,^ ^14^ ^,^ ^16^, can be absorbed in the area of the lesion and maintain concentration in the blood. 

 Through this study, it can be proved that the absorption of metronidazole occurs. Nevertheless, the circulating levels achieved in the various groups were not correlated directly and significantly with the concentration used or the time of use, which favors the local use of metronidazole in higher concentrations and for prolonged times. 

 In studies with experimental animals, metronidazole solutions at a dosage of 20mg/kg resulted in delayed healing^16^. A similar effect was reported for a dosage of 160mg/kg^13^. Dosages higher than 20 and lower than 160mg/kg promoted an increase of granulation tissue and epithelialization^10^ ^,^ ^12^ ^-^ ^15^.^ ^ It may be that dosages up to 20mg/kg are not sufficient to facilitate healing, and dosages higher than 160mg/kg may have compromising absorption, leading to delayed healing. 

 It is also possible to consider that if the dosage is proportional to the healing stimulus, provided it is not at a toxic level, higher dosages would lead to better results in terms of healing. It is important to consider that the larger the area of the wound, the more likely it is that its absorption capacity will be greater. 

 Although in this study metronidazole concentrations were found in the blood, these concentrations were very low. The concentrations found on the third, seventh and fourteenth days were approximately 0.28 mg/l and irrespective of the concentration applied to the wounds of the rats. This corroborates the report of high concentrations of metronidazole (25%), used in periodontal pockets, not having resulted in high plasma concentrations^18^. It is an important observation that in periodontal pockets, with intact tissue, and open wounds, as in the model applied in this study, the metronidazole concentration in the blood was low. 

 The total blood volume of the rat is equal to 6.4% of its total weight, being 58 to 70ml/kg. Considering that the rats used in this study weighed between 300 and 350g, their total blood volumes varied from 17.4ml to 24.5ml. As the concentrations found in the experimental group with 12% metronidazole varied from 0.2323mg/ml to 0.2709mg/ml, considering the weight of the animals, we had a detected presence of 0.004g to 0.007g of metronidazole in the bloodstream, corresponding in terms of the total weight of the rats to between 0.001% and 0.002% of their weight. This characterizes an extremely low level of metronidazole in the bloodstream.^^

^^Absorption was similar regardless of the concentration used. There is likely to be a limit to the absorption capacity. It may be related to the extent of the area to which it is applied. Considering that the lesions had similar dimensions in all the animals, it is possible to suppose that there is a limit for the absorption. and that the concentration in the blood is related to the extent of the lesion.^^

^^The present study is in keeping with other studies that indicate the possibility of using high concentrations of metronidazole topically without inducing high plasma concentrations^18^. 

## Conclusion

 The topical application of metronidazole on experimental full-thickness skin, wiht similar area extent promotes similar absorption, with the detection of low concentrations in the bloodstream irrespective of the concentration of the solution applied and the period of time in which it was applied.
